# Top 100 most-cited articles on tau protein: a bibliometric analysis and evidence mapping

**DOI:** 10.3389/fnins.2024.1345225

**Published:** 2024-01-31

**Authors:** Zheping Chen, Guoliang Shan, Xinyue Wang, Yaqun Zuo, Xinyu Song, Yufeng Ma, Xin Zhao, Yanwu Jin

**Affiliations:** Department of Anesthesiology, The Second Hospital, Cheeloo College of Medicine, Shandong University, Jinan, China

**Keywords:** tau protein, bibliometric analysis, top-cited, CiteSpace, VOSviewer

## Abstract

**Background:**

Tau, a microtubule-associated protein extensively distributed within the central nervous system (CNS), exhibits close associations with various neurodegenerative disorders. Here, we aimed to conduct a qualitative and quantitative bibliometric study of the top 100 most-cited publications on tau protein and reveal the current research hotspots and future perspectives.

**Methods:**

The relevant literature was retrieved from the Web of Science Core Collection. CiteSpace (v6.2.R4) and VOSviewer (1.6.19) were adopted for bibliometric analysis with statistical and visual analysis.

**Results:**

Citations per article ranged from 615 to 3,123, with a median number of 765.5 times. “Neuroscience” emerged as the most extensively researched subject in this field. The USA has emerged as the leading country, with a publication record (*n* = 65), total citations (*n* = 66,543), strong centrality (0.29), and extensive international collaborations. Harvard University (*n* = 11) and the University of California, San Francisco (*n* = 11) were the top two institutions in terms of publications. *Neuron* dominated with 13 articles in the 37 high-quality journals. M. Goedert from the MRC Laboratory of Molecular Biology was the most productive (*n* = 9) and top co-cited (*n* = 179) author. The most frequently studied keywords were Alzheimer’s disease (*n* = 38). Future research is anticipated to intensify its focus on the pathogenesis of various tau-related diseases, emphasizing the phosphorylation and structural alterations of tau protein, particularly in Alzheimer’s disease.

**Conclusion:**

The pathogenesis of various tau-related diseases, including the phosphorylation and structural alterations of the tau protein, will be the primary focus of future research, with particular emphasis on Alzheimer’s disease as a central area of investigation.

## 1 Introduction

Microtubule-associated protein Tau (Tau) was first isolated from pig brains in 1975 by the Weingarten MD Group and named “Tau” for its function in promoting microtubule formation (Weingarten et al., 1975). Under physiological homeostasis, tau protein exerts its function by facilitating tubulin assembly and augmenting microtubule stability (Holper et al., 2022; Hu et al., 2023; Jiménez, 2023). However, under certain pathological conditions, tau protein may form paired helical filaments (PHFs) and neurofibrillary tangles (NFTs) through aggregation (Ossenkoppele et al., 2022). These structures have been documented in many neurological diseases collectively termed “tauopathy,” including Alzheimer’s disease (AD) (Seidler et al., 2022; Chang et al., 2024), Pick disease (PD) (Schweighauser et al., 2023), progressive supranuclear palsy (PSP) (Darricau et al., 2023), frontotemporal dementia with parkinsonism-17 (FTDP-17), and so on (Langworth-Green et al., 2023; Wang et al., 2023). AD is the most extensively researched among these conditions, and as a leading cause of dementia and mortality in older adults, it has emerged as a major public health concern and challenge globally (Mattsson-Carlgren et al., 2023; Mummery et al., 2023). Despite recent advancements in comprehending the physiological and pathological role of tau protein, the mechanisms underlying tau pathology and tau-mediated neurodegeneration remain poorly understood, warranting further research.

Bibliometric analysis has become an indispensable tool in medical research due to its ability to provide a quantitative evaluation of scientific publications (Jain et al., 2023; Kokol, 2023; Sayegh et al., 2023). This method involves the use of mathematical and statistical techniques to analyze various aspects of scholarly literature. The number of citations in an article serves as a valuable metric to assess the influence and significance it holds within the scientific community (Shekhani et al., 2017). Citations provide researchers with a quantitative measure of how extensively their work has been referenced by other scholars in their own studies, indicating its impact on advancing knowledge and shaping future research directions (Merigó and Núñez, 2016; Huang et al., 2022; Kuang et al., 2023).

To the best of our knowledge, no bibliometric analysis has hitherto focused on tau protein. Herein, we conducted a bibliometric analysis to examine the research topics and trends in the field of tau protein to assist scholars in refining their research focus, enabling them to investigate tau protein within a specific domain and analyze research trends over a defined timeframe, thereby uncovering new findings within this domain.

## 2 Methods

### 2.1 Data sources and search strategies

Bibliometric data about tau protein were obtained from the Science Citation Index Expanded (SCIE; 1900–2023) in the Web of Science Core Collection (WoSCC) database within a single day (20 September 2023) to mitigate potential bias arising from daily database updates, using the search formula: TS = (tau protein) OR TS = (tau proteins) OR TS = (microtubule-associated protein tau) OR TS = (tauopathy) OR TS = (tauopathies), with the period of publication ranging from 1900 to 2023. Only articles and reviews were included in the analysis. Abstracts, editorials, proceeding papers, book chapters, articles, and retracted publications were excluded. A total of 33,096 publications were retrieved from the WoSCC. Articles were ranked based on the total number of citations. In cases where articles had an equal number of citations, the more recent ones were given a higher ranking.

### 2.2 Inclusion and exclusion criteria

The publications were ranked in descending order based on their overall “citation frequency.” Two independent reviewers initially screened the articles by reading the full text of the article to confirm whether it was related to tau protein. In cases of disagreement between two researchers regarding whether an article should be included or not, a third researcher was consulted to resolve any discrepancies through consensus discussions. The exclusion criteria were as follows: (1) studies not related to tau protein; (2) studies that did not focus on tau protein; (3) studies not written in English; and (4) studies not described in an original article or review.

### 2.3 Statistical analysis and visualization

The data about the top 100 publications on tau protein were then converted to txt format and exported for bibliometric analysis using CiteSpace (6.2.R4) and VOSviewer (1.6.19). CiteSpace is a visualization software based on the Java platform developed by Professor Chaomei Chen to effectively identify prominent scientific institutions, authors, keywords, research trends, and hotspots (Chen, 2004). Each node in the visualization map generated by CiteSpace represents a country, institution, author, or keyword. The frequency of occurrence or citation is represented by the size of nodes, while different years are indicated by the color of nodes. The thickness of a colored circle indicates the degree of centrality. The nodes exhibiting high centrality (>0.1) were commonly regarded as pivotal or critical points in a field. CiteSpace parameters were set as follows: time slicing from January 1977 to December 2019 (years per slice = 1), node types selecting (country, institution, author, or keyword), selection criteria selecting *g*-index (*k* = 25), and setting the others as default values. Keyword clustering was conducted using a new semi-automated synthesis approach, synthetic knowledge synthesis (SKS), using VOSviewer (Kokol, 2023). The visual elements in VOSviewer’s maps are determined by the number of projects and the strength of cooperation and co-occurrence, with node and line sizes corresponding to these factors (van Eck and Waltman, 2010). The SKS approach is a novel synthesis approach based on the triangulation of distant reading, bibliometric mapping, and content analysis, facilitating the execution of a wide range of mapping functions and providing comprehensive quantitative and qualitative evidence to support research findings (Zagoranski et al., 2021; Kokol, 2023).

The impact factor (IF) of journals is based on the Journal Citation Report (2022).^1^ A visual network analysis of collaborative efforts between countries was generated by Scimago Graphica (1.0.36). In addition, the bibliometrix package in R programming language (4.2.3) was used to generate maps illustrating the production of top authors over time, the most co-cited references, and a geographical map depicting the countries/regions involved in tau protein research.

## 3 Results

### 3.1 Characteristics of the included articles

**Figure 1:**
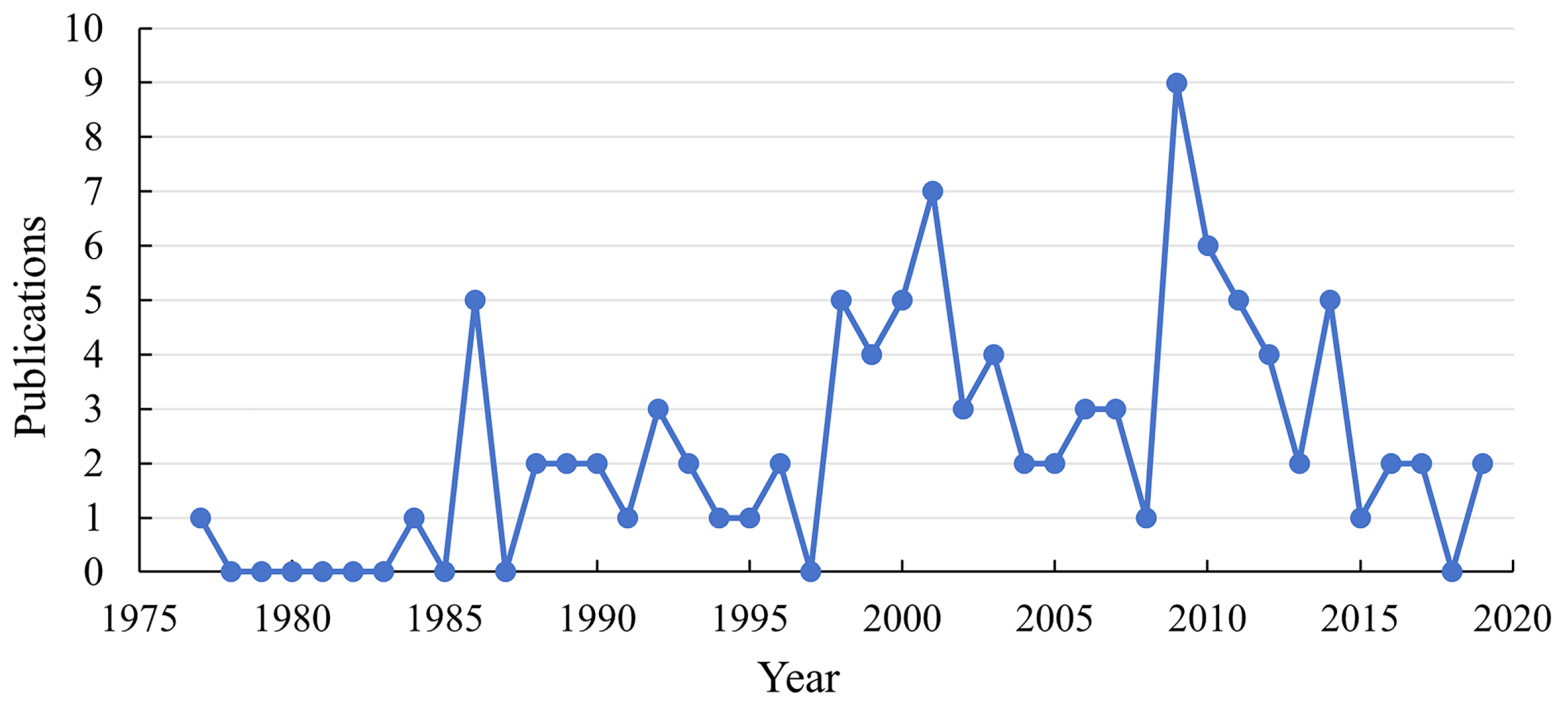
Trend chart of the annual number of published articles in the top 100 most-cited articles.

We retrieved the top 100 most-cited articles related to tau protein from the WoSCC database and ranked them in descending order by ranking of citations. Of the 100 papers, 86 were articles, and 14 were reviews. Table 1 shows the top 10 most-cited articles on tau protein. As shown in Figure 1, these publications were published between 1977 and 2019, ranging from 0 to 9 articles annually. The top 100 most-cited articles were cited a total of 99,008 times, and citations per article ranged from 615 to 3,123, with a median number of 765.5 times. The highest number of top 100 most-cited articles was published in 2009 (*n* = 9), followed by 2001 (*n* = 7). Only two studies were cited more than 3,000 times, five articles were cited more than 2,000 times, and one-third of the articles (*n* = 34) were cited more than 1,000 times. A total of 574 authors from 29 countries/regions, representing 208 institutions and contributing to 37 journals, participated in this comprehensive study.

**Table 1:** Top 10 most-cited articles on tau protein.

Rank	Article title	Journal	Publication year	IF	TC	ACY
1	Triple transgenic model of Alzheimer’s disease with plaques and tangles: Intracellular Aβ and synaptic dysfunction	*Neuron*	2003	16.2	3,123	148.71
2	Abnormal phosphorylation of the microtubule-associated protein-tau (Tau) in Alzheimer cytoskeletal pathology	*PNAS*	1986	11.1	3,001	78.97
3	Association of missense and 5′-splice-site mutations in tau with the inherited dementia FTDP-17	*Nature*	1998	64.8	2,781	106.96
4	Clinical and biomarker changes in dominantly inherited Alzheimer’s disease	*NEJM*	2012	158.5	2,429	202.42
5	Neurodegenerative tauopathies	*Annual Review of Neuroscience*	2001	13.9	2,064	89.74
6	Multiple isoforms of human microtubule-associated protein tau: Sequences and localization in neurofibrillary tangles of Alzheimer’s disease	*Neuron*	1989	16.2	1,953	55.80
7	Staging of Alzheimer disease-associated neurofibrillary pathology using paraffin sections and immunocytochemistry	*Acta Neuropathologica*	2006	12.7	1,936	107.56
8	Tau-mediated neurodegeneration in Alzheimer’s disease and related disorders	*Nature Reviews Neuroscience*	2007	34.7	1,669	57.55
9	Chronic traumatic encephalopathy in athletes: Progressive tauopathy after repetitive head injury	*Journal of Neuropathology and Experimental Neurology*	2009	3.2	1,535	102.33
10	Mutations in progranulin cause tau-negative frontotemporal dementia linked to chromosome 17	*Nature*	2006	64.8	1,521	84.50

IF, impact factor; TC, total citations; ACY, average citation by year; PNAS, The Proceedings of the National Academy of Sciences; NEJM, The New England Journal of Medicine.

### 3.2 Research direction

According to WoSCC categories, the top 100 most-cited articles on tau protein were classified into various research topics (Table 2), with “Neurosciences” (*n* = 35) emerging as the most important research path, followed by “Multidisciplinary Sciences” (*n* = 31), “Clinical Neurology” (*n* = 19), and “Biochemistry Molecular Biology” (*n* = 15).

**Table 2:** WOS categories in the top 100 cited papers on tau protein.

Web of science category	Record count
Neurosciences	35
Multidisciplinary Sciences	31
Clinical Neurology	19
Biochemistry Molecular Biology	15
Cell Biology	13
Pathology	9
Genetics Heredity	3
Medicine General Internal	3
Medicine Research Experimental	3
Biophysics	1
Physiology	1

### 3.3 Analysis of countries/regions

**Figure 2:**
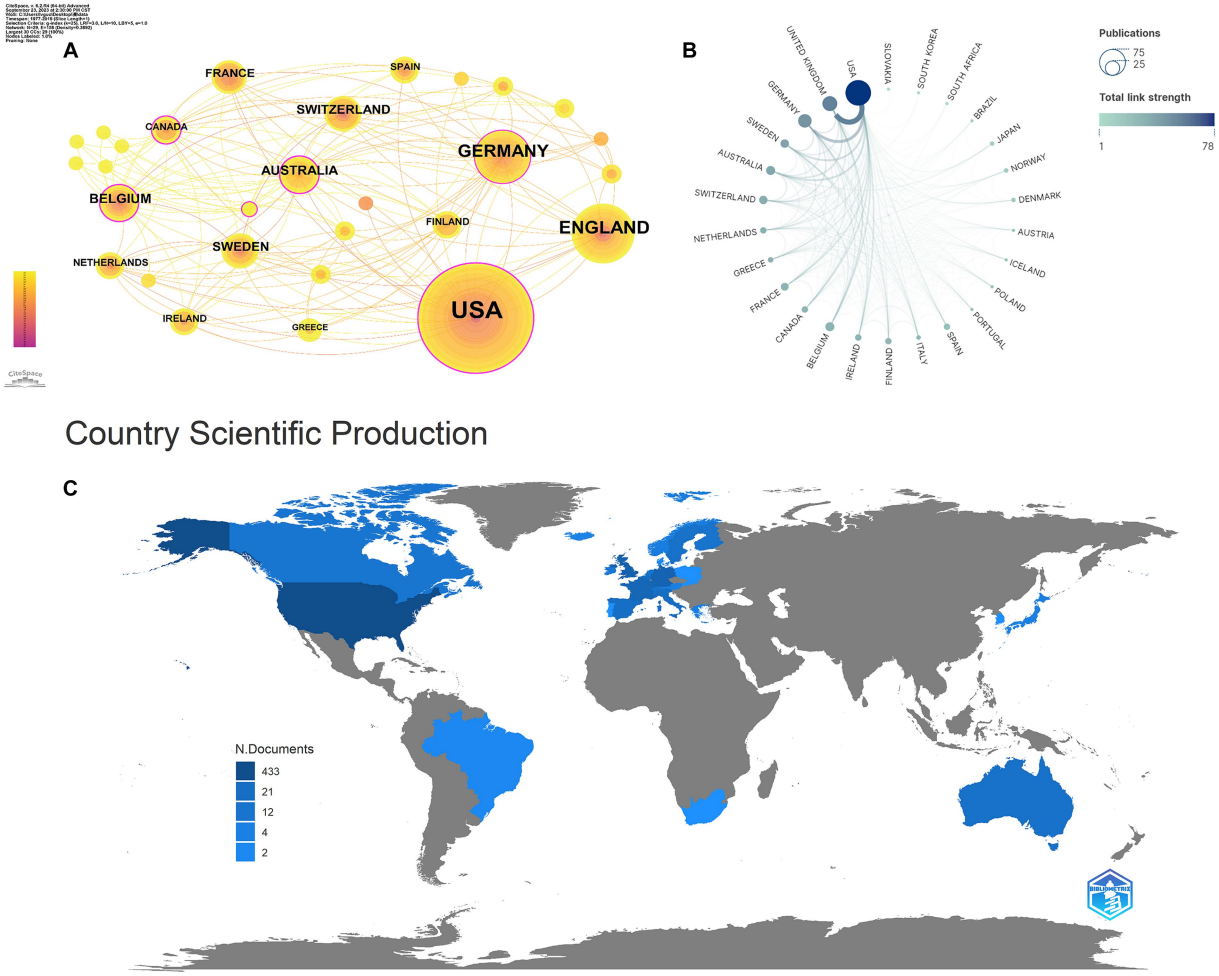
Cooperation between countries/regions. **(A,B)** The cooperation networks between different countries/regions contributed to the top 100 highly cited articles; **(C)** the geography map of countries/regions for tau protein.

As shown in Table 3, in total, 29 countries/regions contributed to the top 100 most-cited articles, with the USA being the leading contributor in terms of publications (*n* = 65), followed by the UK (*n* = 21) and Germany (*n* = 18). The USA (*n* = 66,534), the UK (*n* = 24,070), and Germany (*n* = 16,509) emerged as the leading countries in terms of citations. In addition, according to node centrality analysis by CiteSpace, the USA had the highest centrality (centrality = 0.29), which was relatively higher than other countries, indicating that the USA plays a prominent role in this field as well as having extensive cooperation with other countries (Figures 2A–C).

**Table 3:** Top 5 countries/regions of top 100 most-cited articles on tau protein.

Rank	Country/region	Count	Centrality	Citation
1	The USA	65	0.29	66,543
2	The UK	21	0.07	24,070
3	Germany	18	0.13	16,509
4	Australia	8	0.25	11,458
5	Belgium	8	0.21	7,443

### 3.4 Analysis of institutions

**Figure 3:**
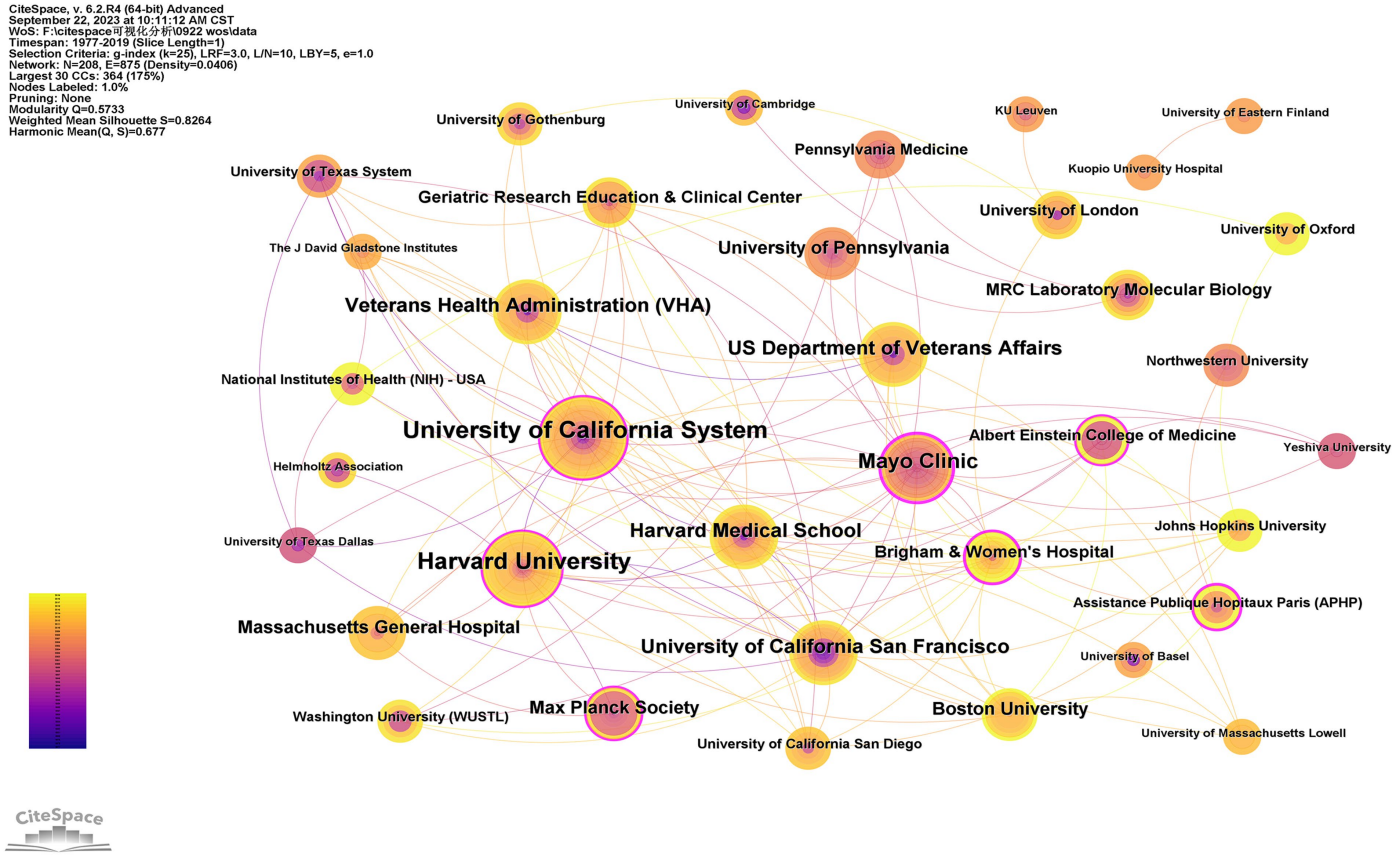
Institutions of the top 100 most-cited articles on tau protein.

A total of 208 institutions contributed to the top 100 most-cited articles, and the top two institutions with publications were Harvard University (*n* = 11) and the University of California, San Francisco (*n* = 11). The top two institutions were Mayo Clinic (*n* = 11,707) and Harvard University (*n* = 10,409) in terms of citations (Table 4). The Mayo Clinic had the highest centrality (centrality = 0.46), indicating the dominance of institutions from the USA in the field of tau protein (Figure 3).

**Table 4:** Top eight institutions of the top 100 most-cited articles on tau protein.

Rank	Institution	Count	Centrality	Citation	Country
1	Harvard University	11	0.14	10,409	The USA
2	University of California, San Francisco	11	0.18	9,090	The USA
3	Mayo Clinic	8	0.46	11,707	The USA
4	University of Pennsylvania	7	0.03	9,446	The USA
5	Washington University	6	0.01	8,128	The USA
6	Boston University	6	0.03	6,173	The USA
7	MRC Laboratory of Molecular Biology	5	0.04	3,508	The UK
8	Max Planck Research Unit for Structural Molecular Biology	5	0.11	6,101	Germany

### 3.5 Analysis of journals

**Figure 4:**
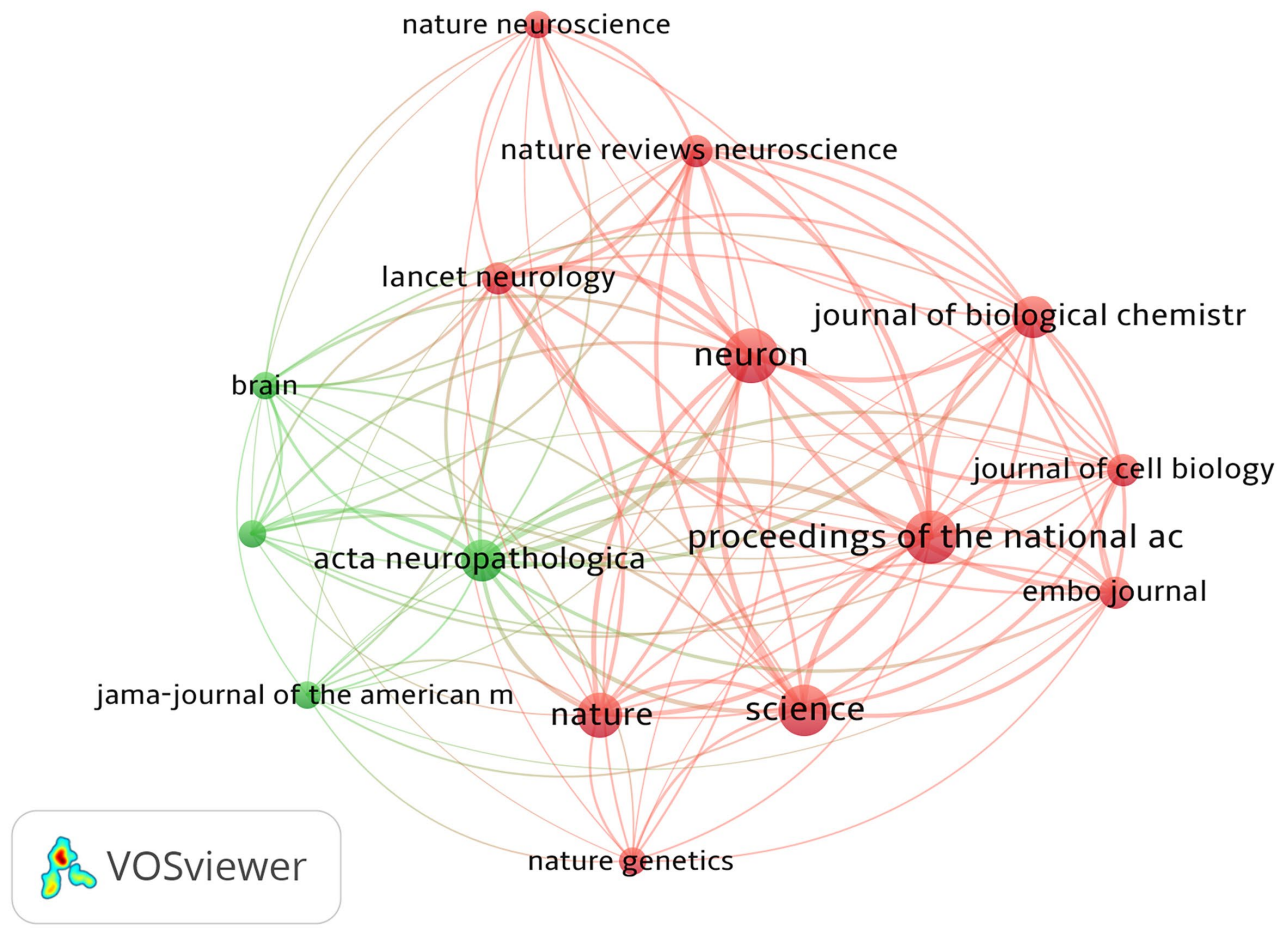
Journal analysis of the top 100 most-cited articles on tau protein.

The top 100 most-cited articles were distributed across 37 journals. As shown in Table 5 and Figure 4, the top journal for citations was *Neuron* (*n* = 13,207). *Neuron* published the most articles (*n* = 13), followed by *PNAS* (*n* = 12), *Science* (*n* = 11), and *Nature* (*n* = 8). These four journals belong to Q1 according to the JCR categories; 44% of articles in the top 100 were published in these four journals, all of which hold commendable academic standing. The aforementioned findings suggest that these top four authoritative journals serve as primary sources of information regarding the latest advancements in tau protein research.

**Table 5:** Top six journals with the most-cited articles on tau protein.

Rank	Journal	Count	Citation	IF (2022)	JCR (2022)
1	*Neuron*	13	13,207	16.2	Q1
2	*PNAS*	12	11,832	11.1	Q1
3	*Science*	11	10,821	56.9	Q1
4	*Nature*	8	9,435	64.8	Q1
5	*Acta Neuropathologica*	6	5,524	12.7	Q1
6	*Journal of Biological Chemistry*	6	5,138	4.8	Q2

PNAS, The Proceedings of the National Academy of Sciences.

### 3.6 Analysis of authors and co-cited author

**Figure 5:**
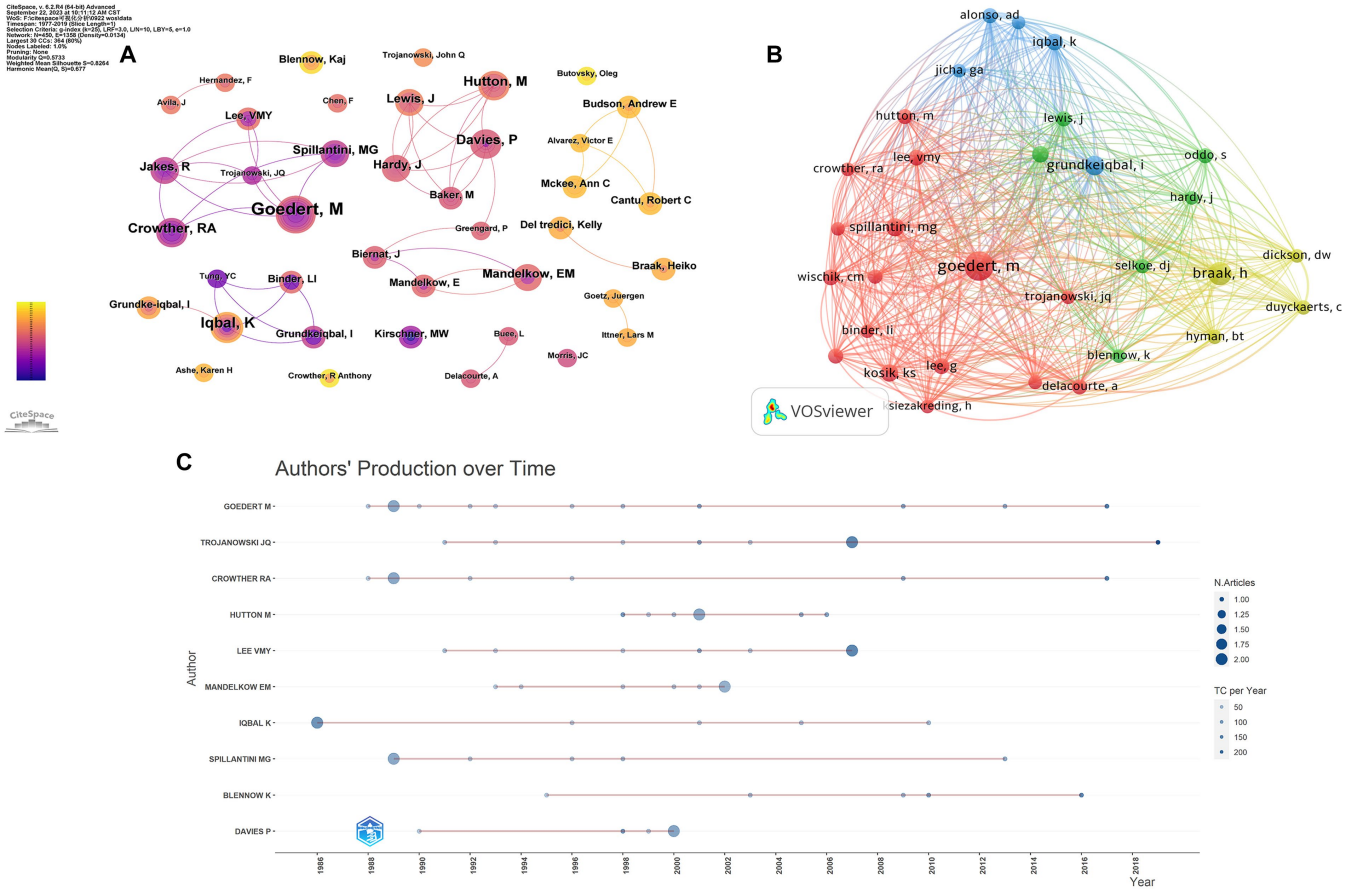
Authors’ analysis of tau protein. **(A)** Authors’ analysis; **(B)** co-cited authors’ analysis; **(C)** top-cited authors’ production over time.

A total of 364 authors were involved in the top 100 most-cited articles in the field of tau protein, with eight authors contributing more than five publications. M. Goedert from the MRC Laboratory of Molecular Biology was the top contributor with nine articles and the top-cited author with 10,369 citations (Figure 5A). Furthermore, M. Goedert was the top co-cited author with 179 citations. In Figure 5B, the node representing M. Goedert was the largest, significantly surpassing others. In summary, M. Goedert not only ranked as the top productive author among the 100 articles but also stood out as the most co-cited author, affirming widespread recognition as an expert in the field of tau protein. Figure 5C illustrates the top 10 authors and their publications over time. The size of each circle corresponds to the number of articles published, with a positive correlation. Additionally, the intensity of color is directly proportional to the number of citations.

### 3.7 Analysis of keywords

#### 3.7.1 Keyword co-occurrence

**Figure 6:**
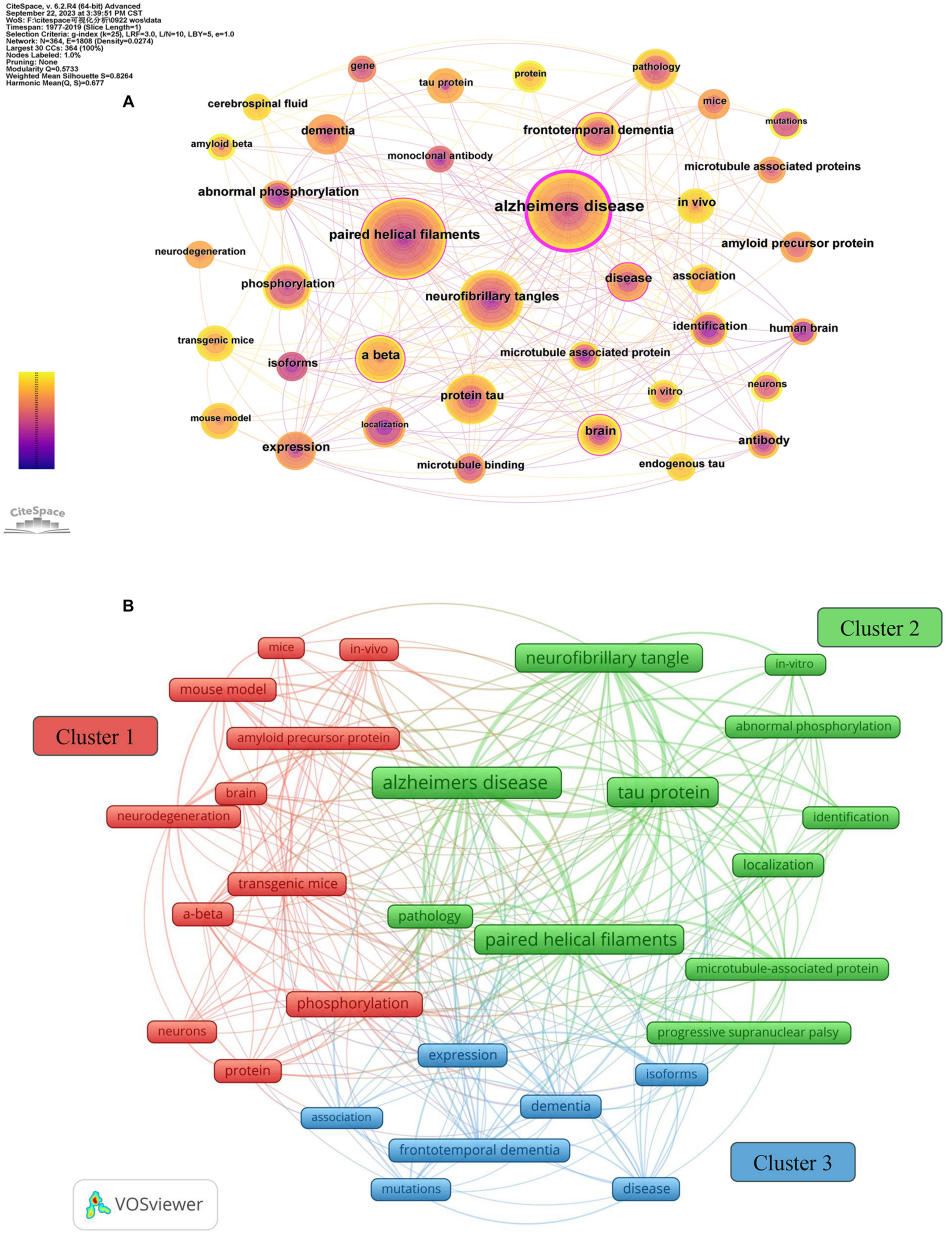
Keyword co-occurrence analysis of tau protein. **(A)** The network map of the co-occurrence keywords; **(B)** clusters of keywords.

Keywords play a crucial role in providing readers with a quick and concise understanding of the main themes and ideas discussed in an article. The top 100 most-cited articles included in this study comprised 364 keywords. In addition to the search terms, the high frequency of occurrence was “Alzheimer’s disease” (*n* = 38), “paired helical filaments” (*n* = 34), “neurofibrillary tangles” (*n* = 27), “phosphorylation” (*n* = 16), and “Aβ” (*n* = 11). Node centrality analysis demonstrated that the top keyword was “Alzheimer’s disease” (centrality = 0.69). The results revealed that researchers predominantly focused on the molecular structure and phosphorylation of tau protein, as well as the involvement of Aβ and tau protein in AD (Figure 6A).

#### 3.7.2 Clusters of keywords

Clustering analysis of keywords, employing the SKS approach with VOSviewer, demonstrated separation into three primary clusters (Kokol, 2021; Zagoranski et al., 2021). Based on node colors, these clusters are outlined as follows: cluster 1 (red) involves investigating Aβ and phosphorylated tau protein in animal models through *in vivo* experiments to unravel the pathogenesis of neurodegenerative disorders; cluster 2 (green) centers around the examination of tau protein through *in vitro* experiments to comprehend the pathogenesis of AD and PSP; cluster 3 (blue) encompasses tau-related diseases and associations. The results emphasize that exploring tau-related diseases and their underlying mechanisms, particularly through *in vitro* and *in vivo* experiments, especially those involving Aβ and tau protein, represents significant research inquiries in this field. There is an emphasis on AD as a central area of investigation (Figure 6B; Table 6).

**Table 6:** Clusters of keywords and research topic.

Cluster color	Representative keywords	Research topic
Red (11 keywords)	Phosphorylation (*n* = 16), Aβ (*n* = 11), transgenic mice (*n* = 10), protein (*n* = 10), mouse model (*n* = 9), *in vivo* (*n* = 8), neurodegeneration (*n* = 7), amyloid precursor protein (*n* = 6), brain (*n* = 6), neurons (*n* = 6), mice (*n* = 5)	Investigation of Aβ and phosphorylated tau protein in animal models via *in vivo* experimentation to elucidate the pathogenesis of neurodegenerative disorders
Green (11 keywords)	Alzheimer’s disease (*n* = 38), paired helical filaments (*n* = 34), tau protein (*n* = 31), neurofibrillary tangle (*n* = 27), pathology (*n* = 12), localization (*n* = 9), microtubule-associated protein (*n* = 8), identification (*n* = 7), progressive supranuclear palsy (*n* = 7), abnormal phosphorylation (*n* = 6), *in vitro* (*n* = 5)	Investigation of tau protein via *in vitro* experimentation to elucidate the pathogenesis of Alzheimer’s disease and progressive supranuclear palsy
Blue (7 keywords)	Frontotemporal dementia (*n* = 11), dementia (*n* = 10), disease (*n* = 9), expression (*n* = 9), mutations (*n* = 6), isoforms (*n* = 6), association (*n* = 5)	Tau-related diseases and association

#### 3.7.3 Timeline of keywords

**Figure 7:**
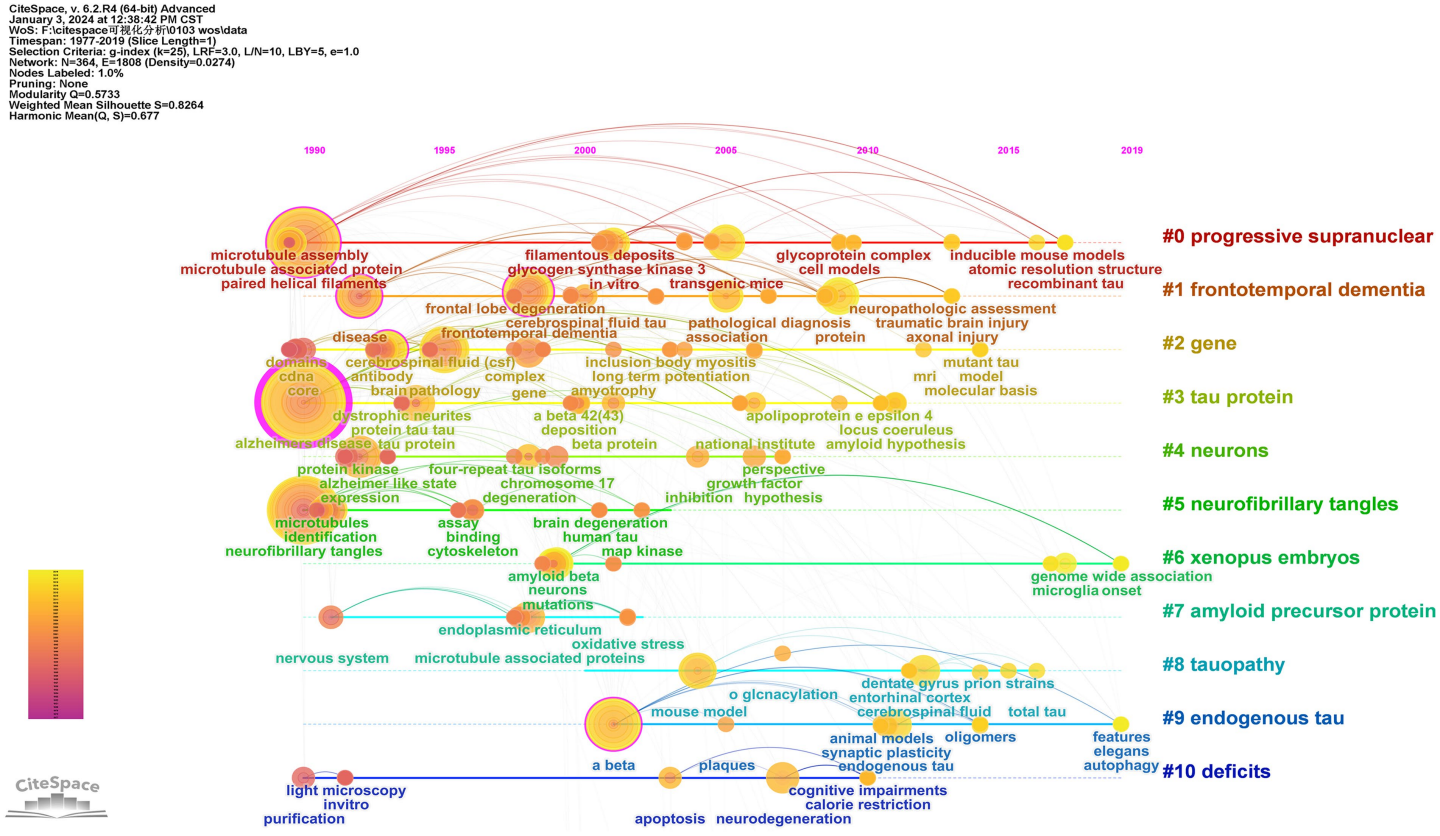
Timeline view of keyword analysis of tau protein.

CiteSpace employed the timeline as the analysis node to generate a keyword time graph depicting the temporal evolution of keywords. This timeline view offers a clearer visualization of historical research results, trends, and internal relationships within each cluster. Each node represents a distinct keyword, with larger nodes indicating a higher frequency of occurrence. The gradual transition in line color from cool to warm tones signifies the changing trend in keyword appearance over time. Examining the time development of keywords, in 1990, high-frequency keywords such as “Alzheimer’s disease,” “paired helical filament,” and “neurofibrillary tangle” indicated the early initiation of research with substantial outcomes. From 1991 to 2018, extensive research covered diverse topics, including “protein tau,” “phosphorylation,” “Aβ,” “frontotemporal dementia,” “pathology,” “expression patterns,” “neurodegenerative diseases,” and “cognitive impairments.” Currently, the forefront of significant research involves “autophagy” and “genome-wide association (GWAS),” suggesting promising avenues for future scholarly investigations (Figure 7).

### 3.8 Analysis of co-cited references

**Figure 8:**
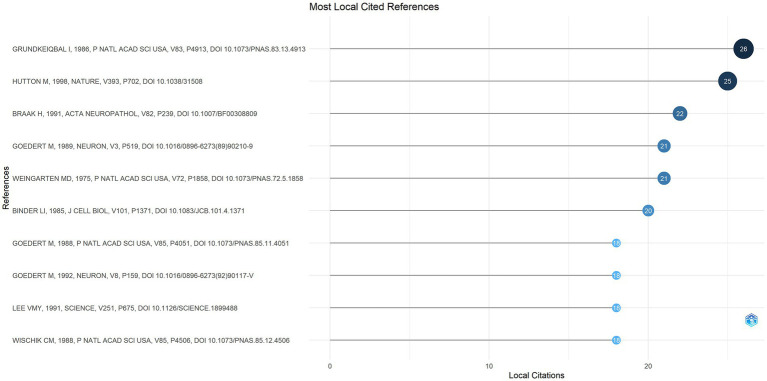
Co-cited references analysis of the top 100 most-cited articles on tau protein.

A total of 4,554 references were cited by the top 100 most-cited articles included in this study. “Abnormal phosphorylation of the microtubule-associated protein tau (tau) in Alzheimer cytoskeletal pathology,” published by I. Grundke-Iqbal in *PNAS*, was the most co-cited article with 26 citations (Figure 8). The study revealed that the tau protein in the Alzheimer’s brain is an abnormally phosphorylated constituent of PHFs.

## 4 Discussion

It is of utmost importance for researchers to discern high-quality and meaningful research amidst the extensive body of published literature within a specific field. Tau protein represents a significant research topic in neuroscience, and comprehensively analyzing all articles published in this field over the past decade poses a challenge, given the potential annual publication count exceeding 5,000. While bibliometric analysis within a defined timeframe can effectively elucidate research trends, the expansive nature of tau protein research complicates the identification of distinct focal points. Our study aims to identify the most crucial research areas within the tau protein field, laying a foundational framework for subsequent investigations into specific facets of tau research spanning the past decade. Serving as the inaugural bibliometric analysis of the top 100 most-cited articles and influential works in the tau protein domain, this study unveils current research hotspots and future perspectives by scrutinizing data on countries/regions, institutions, authors, journals, and keywords, shedding light on potential future research directions.

### 4.1 General information from the top 100 cited articles

Our findings indicate that the median number of citations for the top 100 most-cited articles on tau protein was 765.5, significantly surpassing those in other fields such as anaphylaxis, PD-L1, and bladder cancer. This discrepancy underscores tau protein’s status as a current research hotspot (Mainwaring et al., 2020; Song Y. et al., 2023). The substantial variance in citation numbers implies that studies related to tau protein have significantly contributed to advancing our understanding of neurodegenerative diseases. The heightened interest in tau protein research may be attributed to recent breakthroughs or advancements in this field (Luciani et al., 2023; Zhang Z. Y. et al., 2023; Singh et al., 2024). Scientists have recently unveiled novel mechanisms or identified promising targets associated with tau pathology that could potentially revolutionize our understanding and treatment approaches for neurodegenerative diseases (Kim et al., 2023; Kyalu Ngoie Zola et al., 2023; Langworth-Green et al., 2023). Moreover, it is noteworthy that high citation counts not only reflect scientific interest but also establish credibility within the academic community, serving as a fundamental pillar of research in this field (Nielsen and Andersen, 2021; Radosic and Diener, 2021). In terms of article type, more than 80% of the top 100 cited papers were original articles.

The peak number of top 100 cited articles occurred in 2009, while the most concise span of publications was observed in 2019. In accordance with the WoSCC classification, our analysis revealed that “neuroscience” stood out as the most extensively researched subject in this field. This observation underscores the increasing interest and significance attached to comprehending the role of tau protein in the brain and central nervous system (CNS).

A total of 29 countries/regions and 208 institutions were involved in the publication of the top 100 cited articles on tau protein. The USA stands out as the leading country in tau protein research, boasting a substantial publication record, high total citations, strong centrality, and extensive international collaborations. Among the top eight institutions, six are based in the USA, including Harvard University. This dominance underscores the influential position of the USA in the field, likely attributed to its robust research and development infrastructure, substantial investment in scientific research and innovation, and a commitment to staying at the forefront of technological advancements, fostering an environment conducive to groundbreaking discoveries.

The top 100 cited articles were dispersed among 37 journals, with *Neuron* being the top journal in terms of citations. *Neuron* published the highest number of articles, followed by *PNAS*, *Science*, and *Nature*. These four journals are categorized as Q1 according to the JCR classifications, and 44% of the top 100 articles were published in these leading journals. Their recognized contributions to advancing knowledge and fostering intellectual exchange within the academic community suggest that these journals are likely to continue publishing influential work in this field in future.

Analysis of authors and co-cited authors has revealed that M. Goedert from the MRC Laboratory of Molecular Biology not only ranked as the top productive author in the top 100 most-cited articles but also emerged as the top co-cited author, widely recognized as an expert in the field of tau protein. The most-cited article, titled “Triple transgenic model of Alzheimer’s disease with plaques and tangles: Intracellular Aβ and synaptic dysfunction,” elucidates the pathogenesis of AD in mice, highlighting that Aβ deposits precede tau alterations and result in changes in long-term synaptic plasticity (Oddo et al., 2003). The most co-cited article, “Abnormal phosphorylation of the microtubule-associated protein tau (tau) in Alzheimer cytoskeletal pathology,” posits that tau, as an abnormally phosphorylated protein component of paired helical filaments, is implicated in the brain pathology of AD (Grundke-Iqbal et al., 1986). These findings underscore the enduring dominance of tau protein research related to AD, attracting extensive attention from researchers. The importance of studying tau protein lies in its potential as a therapeutic target for treating or preventing AD. By comprehending the mechanisms underlying its pathology, researchers aim to develop interventions capable of halting or slowing down disease progression (Congdon and Sigurdsson, 2018; Chang et al., 2021). Moreover, investigating tau protein provides insights into other neurodegenerative disorders beyond AD (Iqbal et al., 2016; Josephs, 2017). Similar pathological changes involving the abnormal accumulation of misfolded proteins have been observed in diseases like FTD and PSP (Spillantini and Goedert, 2013; Dam et al., 2021; Kouri et al., 2021; Chouliaras et al., 2022). Therefore, the investigation of tau protein in AD serves as a fundamental pillar of research across related disciplines.

### 4.2 Research hotspots

Keyword co-occurrence analysis serves to uncover relationships between keywords in a collection of publications, revealing distinct areas of research focus and subjects. In our study, we made a noteworthy observation regarding the clustering of keywords using the SKS approach. The SKS approach, a novel knowledge synthesis methodology based on the triangulation of distant reading, bibliometric mapping, and content analysis, facilitates a wide range of mapping functions. It provides comprehensive quantitative and qualitative evidence to support research findings (Kokol, 2021, 2023).

Our clustering analysis highlighted three main aspects. First, there is a strong emphasis on Aβ and phosphorylated tau protein in animal models through *in vivo* experimentation to understand the pathogenesis of neurodegenerative disorders. Researchers aim to elucidate the pathogenesis of these conditions by studying these proteins within living organisms. Through careful observation and analysis, scientists can examine the interactions of Aβ and phosphorylated tau protein with neural cells, contributing to the progression of neurodegenerative disorders. Investigating the effects of Aβo-neurotoxicity and pathologic tau accumulation on neuronal function and connectivity may reveal potential therapeutic targets to mitigate or reverse cognitive decline associated with diseases (Stoner et al., 2023; Li et al., 2024; Zhang et al., 2024). Zhao et al. (2023) found that the administration of rFOXN1 can reduce Aβ plaque load and phosphorylated tau in the brain, improving cognitive performance. Second, the investigation of tau protein through *in vitro* experimentation to understand the pathogenesis of AD and progressive supranuclear palsy (PSP) played a significant role in our study. AD and PSP are neurodegenerative disorders associated with abnormal tau protein aggregation (Boxer et al., 2017). Third, there is a pronounced focus on tau-related diseases and associations. The folded form of tau is present in more than 20 neurodegenerative diseases collectively known as tauopathies, including AD, PSP, corticobasal degeneration, argyrophilic grain disease, Pick’s disease, frontotemporal dementia, and others (Shi et al., 2021; Thijssen et al., 2021; Seidler et al., 2022)

From the keyword co-occurrence analysis, it can be inferred that AD receives a higher frequency of investigation compared to other diseases. This increased focus on AD may be attributed to its prevalence and the growing concern of researchers and scientists worldwide. Second, the research focuses on the pathogenesis of Aβ and tau proteins in various diseases. Although the complex Aβ–tau interaction has been partially studied, the specific mechanism remains unclear (Busche and Hyman, 2020; Lee et al., 2022; Vogt et al., 2022).

The evolution of keywords indicates that AD is the central focus of research in this field, with early initiation and fruitful outcomes. Subsequently, extensive investigations have investigated the interaction between tau protein and Aβ across various diseases. In recent years, cutting-edge research has expanded beyond exclusively studying tau protein and Aβ. The focus has shifted toward exploring other crucial aspects related to neurodegeneration. One emerging area is autophagy—a cellular process responsible for clearing out damaged proteins and organelles (Shu et al., 2023). Dysregulation of autophagy has been implicated in several neurodegenerative disorders, including AD (Choi et al., 2023; Mary et al., 2023). Understanding how autophagy contributes to disease pathology may provide new avenues for therapeutic interventions. Recent studies have shown that excessive neuronal C–C chemokine receptor type 5 (CCR5) activation increases autophagy as a cause of tau-related diseases, and pharmacological and genetic inhibition of CCR5 can reduce neurological damage (Festa et al., 2023a,b). Furthermore, GWAS have gained significant attention as they allow researchers to identify genetic variations associated with increased susceptibility or protection against certain diseases. Recently, GWAS have revealed the unique genetic structure of proteins associated with AD (Neumann et al., 2023; Oatman et al., 2023; Ta et al., 2023).

While there is no direct bibliometric analysis focused on tau protein, previous bibliometric analyses of AD have substantiated that tau protein is at the core of AD research, and the research hotspots of AD also encompass tau protein. Fei et al. (2022) found that “circular RNAs,” “regulation of neuroinflammation,” and “tau protein” were the future research directions of non-coding RNAs in AD. Other studies have indicated that tau protein is a keyword associated with glial fibrillary acidic protein (GFAP), mitochondrion, GWAS, and biomarkers in AD (Noda et al., 2023; Song R. et al., 2023; Zhang J. et al., 2023; Zou et al., 2023). Neuroinflammation refers to the activation of immune responses within the CNS, specifically involving glial cells such as microglia and astrocytes. Recent studies have demonstrated that neuroinflammation, particularly involving glial cells, plays a pivotal role in the development of tau pathology, providing valuable insights for future investigations and therapeutic interventions targeting tau in AD (Chen and Yu, 2023; Dutta et al., 2023; Langworth-Green et al., 2023; Lau et al., 2023; Lee et al., 2023).

### 4.3 Future perspectives

The pathogenesis of various tau-related diseases, focusing on the phosphorylation and structural alterations of the tau protein, is anticipated to be a primary area of research in future, with an emphasis on AD as a central investigative focus. The extensive and continually growing body of previous studies on tau protein spans diverse research disciplines, presenting a challenge for researchers in pinpointing specific areas of focus. From our bibliometric analysis, it is evident that AD has become a central topic due to its strong association with the abnormal accumulation and aggregation of tau protein in the brain. Understanding the underlying mechanisms of tau protein pathology has become crucial for the development of effective diagnostic tools, therapeutic interventions, and potential preventive strategies for AD.

In light of the focus on tau research in AD, governments and related agencies have the opportunity to formulate more targeted policies to address this neurodegenerative disorder. Policymakers can enhance support for AD and devise more targeted prevention, diagnosis, and treatment policies to improve disease management and patients’ quality of life. This may involve supporting clinical trials investigating potential drugs or therapies aimed at reducing abnormal tau protein accumulation or promoting its clearance from the brain. Additionally, funding should be directed toward studies exploring non-pharmacological approaches, such as lifestyle modifications (e.g., exercise, diet), which may positively impact tau-related neurodegeneration. To advance research in this field further, funding agencies should not only increase financial support but also encourage interdisciplinary collaborations. Fostering cooperation between researchers across various fields, such as neuroscience, genetics, psychology, and geriatrics, can lead to a more holistic approach to understanding AD.

In future, based on our comprehensive study, we can leverage bibliometric analysis techniques to delve deeper into the application of tau protein in the field of AD over nearly a decade. This extensive analysis will enable us to identify and highlight potential key targets, emerging trends in research drugs, and ongoing clinical trials related to tau protein. By analyzing publication trends over time using bibliometrics, we can track how research on tau protein has evolved within the field of AD. This longitudinal perspective allows us to observe emerging themes or shifts in focus that may indicate new directions for future investigations.

### 4.4 Limitations

In our endeavor to conduct a rigorous bibliometric analysis, certain limitations were inevitably encountered. First, it should be acknowledged that our analysis concentrated exclusively on the top 100 most-cited articles. While this focus facilitated the identification of influential research within these specific articles, it may have inadvertently neglected other valuable literature across various fields. This limitation arises from the potential oversight of lesser-known studies or emerging research that could offer unique insights and perspectives. Second, owing to a limited time frame for publication and fewer citations, high-quality articles published after 2019 might not have been incorporated into our study. Certain publications may remain dormant for an extended period, experiencing a sudden surge in citations, akin to the phenomenon known as “sleeping beauty,” potentially introducing bias (Kokol et al., 2017; Song et al., 2018). Third, not all literature was included in the WoSCC database, raising the possibility of inaccurate representation of citation counts and potential impacts on bibliometric analysis outcomes. Fourthly, the number of citations does not comprehensively reflect research quality. Low citation counts may not necessarily indicate poor research quality, and historically high citation counts may not guarantee long-term influence. Self-citation was not considered, potentially inflating the citation rates of both the cited articles and their respective journals without reflecting their quality. Finally, bibliometric analysis inherently bears limitations, as it primarily assesses quantity, citations, and other indicators of scholarly literature, neglecting critical factors such as research design rationality and experimental method rigor, which contribute to study quality and impact. Moreover, bibliometric analysis may perpetuate a “the rich get richer” phenomenon, wherein already influential research fields or institutions are more likely to receive citations and attention, potentially resulting in biased evaluation outcomes. Despite these limitations, this study, being the first of its kind in the top 100 cited articles on tau protein, provides current status and future directions for further investigation.

## 5 Conclusion

This is the first bibliometric analysis of the top 100 cited articles on tau protein. The predominant research domain for tau protein was identified as *Neuroscience*. The USA emerged as the frontrunner in terms of publication output, total citations, robust centrality, and extensive international collaborations. Harvard University and the University of California, San Francisco secured the top two positions among institutions based on their publication contributions. M. Goedert stood out as the most prolific contributor to the field, holding both the largest number of authored articles and the highest co-citation count. The journal *Neuron* held a dominant position in publishing high-quality articles related to tau protein. The current trajectory of tau protein research predominantly centers around tau-related diseases and the underlying mechanisms within these conditions, with a particular emphasis on the interplay of Aβ and tau protein. Future research is anticipated to intensify its focus on the pathogenesis of various tau-related diseases, emphasizing the phosphorylation and structural alterations of tau protein, particularly in AD.

## Data availability statement

The raw data supporting the conclusions of this article will be made available by the authors, without undue reservation.

## Author contributions

ZC: Writing – original draft. GS: Writing – review & editing. XW: Writing – review & editing. YZ: Writing – review & editing. XS: Writing – review & editing. YM: Writing – review & editing. XZ: Writing – review & editing. YJ: Data curation, Formal analysis, Funding acquisition, Investigation, Methodology, Project administration, Writing – review & editing.

## Footnotes

^1^ https://JCR.Clarivate.com/
